# Phosphate Starvation Triggers Production and Secretion of an Extracellular Lipoprotein in *Caulobacter crescentus*

**DOI:** 10.1371/journal.pone.0014198

**Published:** 2010-12-02

**Authors:** Sophie Le Blastier, Aurore Hamels, Matthew Cabeen, Lionel Schille, Françoise Tilquin, Marc Dieu, Martine Raes, Jean-Yves Matroule

**Affiliations:** 1 Unité de Recherche en Biologie Moléculaire, University of Namur, Namur, Belgium; 2 Department of Molecular, Cellular and Developmental Biology, Yale University, New Haven, Connecticut, United States of America; 3 Unité de Recherche en Biologie Cellulaire, University of Namur, Namur, Belgium; Baylor College of Medicine, United States of America

## Abstract

Life in oligotrophic environments necessitates quick adaptive responses to a sudden lack of nutrients. Secretion of specific degradative enzymes into the extracellular medium is a means to mobilize the required nutrient from nearby sources. The aquatic bacterium *Caulobacter crescentus* must often face changes in its environment such as phosphate limitation. Evidence reported in this paper indicates that under phosphate starvation, *C. crescentus* produces a membrane surface-anchored lipoprotein named ElpS subsequently released into the extracellular medium. A complete set of 12 genes encoding a type II secretion system (T2SS) is located adjacent to the *elpS* locus in the *C. crescentus* genome. Deletion of this T2SS impairs release of ElpS in the environment, which surprisingly remains present at the cell surface, indicating that the T2SS is not involved in the translocation of ElpS to the outer membrane but rather in its release. Accordingly, treatment with protease inhibitors prevents release of ElpS in the extracellular medium suggesting that ElpS secretion relies on a T2SS-secreted protease. Finally, secretion of ElpS is associated with an increase in alkaline phosphatase activity in culture supernatants, suggesting a role of the secreted protein in inorganic phosphate mobilization. In conlusion, we have shown that upon phosphate starvation, *C. crescentus* produces an outer membrane bound lipoprotein, ElpS, which is further cleaved and released in the extracellular medium in a T2SS-dependent manner. Our data suggest that ElpS is associated with an alkaline phosphatase activity, thereby allowing the bacterium to gather inorganic phosphates from a poor environment.

## Introduction

*Caulobacter* species live in all kinds of aquatic environments where nutrients are often scarce. While some nutrients are directly usable by the bacteria, other organic substrates must be partially degraded or modified before being assimilated by the cell as a nutrient source. These processes often rely on degradative enzymes that are secreted at the cell surface or in the extracellular environment depending on specialized secretion systems. The Type II secretion system (T2SS) or main terminal branch of General secretion pathway (Gsp) is well known for its role in virulence of pathogenic species but is also involved in environmental adaptation. A T2SS is present in at least 16 environmental non-pathogenic bacteria [1] where it promotes secretion of enzymes such as lipases in *Acinetobacter calcoceticus* and *Pseudomonas alcaligenes* [2], [3] or chitinase in *Escherichia coli K12* [4]. The T2SS apparatus is composed of at least 12 proteins, and mediates a two-step translocation of secreted proteins across the cell envelope. Briefly, unfolded exoproteins are first exported by the Sec or Tat pathways into the periplasm where they undergo maturation. They are then translocated across the outer membrane through secretin pores [5], [6], [7], [8]. The T2SS was first described in *Klebsiella oxytoca* where it triggers export across the outer membrane of the starch-hydrolysing lipoprotein pullulanase [9]. Pullulanase is a surface anchored lipoprotein partially released in the extracellular environment through the formation of micelles [10]. However, the extracellular release of bacterial surface lipoproteins could occur differently. Recent studies have thus shown that the autotransporter NalP of *Neisseria meningitides* was responsible for the proteolytic cleavage of lactoferrin-binding protein LbpB from the cell surface [11].

Although T2SS-encoding genes are generally well conserved and display a similar genetic organization in many bacterial species, secreted proteins have a wide variety of functions or enzymatic activities [4], [12], [13].

Phosphorus, which can be limiting for *Caulobacter* survival, is present in soil and water in organic and inorganic forms. Inorganic phosphate (P_i_) is the preferential source and is readily taken up by the cell when available. Since nutrient-poor environments can also lack P_i_, many bacterial species have developed mechanisms to use organic phosphates as a source of phosphorus [14]. While some organic phosphates such as dNTPs may be directly incorporated by the cells, others may require extracellular P_i_ release before being taken up [15]. Under phosphate starvation, *C. crescentus* strikingly increases stalk length, thereby optimizing the surface/volume ratio and in turn facilitating phosphate uptake [16], [17]. This process depends on Pst and PhoB [18], which both belong to the *pho* regulon best characterized in *Escherichia coli*. The *pho* regulon [19] also encompasses *phoA*, encoding an alkaline phosphatase required for the utilization of organic phosphates [20]. *C. crescentus* harbors the *pho* regulon but lacks a *phoA* homolog.

In the present study, we report the ability of *C. crescentus* to trigger production and T2SS-dependent secretion of a lipoprotein, ElpS, under phosphate starvation conditions. We show that ElpS is an extracellular outer membrane-anchored protein subsequently released in the environment in a T2SS-dependent manner. Functional data suggest that ElpS is involved in phosphate mobilization and stimulates extracellular alkaline phosphatase activity in low-phosphate medium.

## Results

### *C. crescentus* genome harbours a complete set of T2SS genes

**Figure 1:**
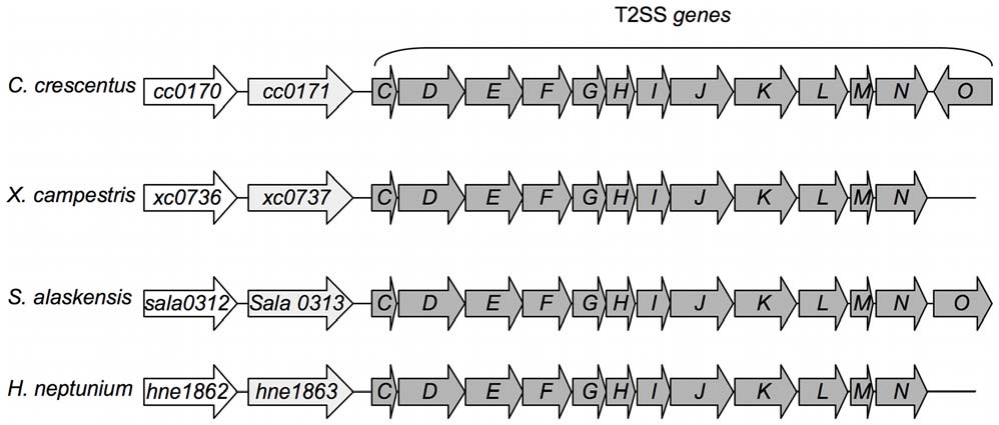
Alignment of genes encoding type II secretion system components and *cc0170*/*cc0171* homologs in individual species. Genes are shown as boxes with arrowheads to indicate their predicted transcriptional orientation. Genes encoding T2SS apparatus are named *C* to *O*. Sequences and alignments were obtained from the CMR website (http://cmr.jcvi.org).

Although T2SS has been largely studied in γ-proteobacteria it appears that many α-proteobacteria possess T2SS genes (e.g., *Bradyrhizobium*, *Gluconacetobacter*, *Mesorhizobium*) [21]. *In silico* analysis of the *C. crescentus* (CB15N) genome reveals the presence of an entire set of T2SS genes, *gspC* (*cc0172*) to *gspN* (*cc0184*) annotated in alphabetical order (Fig. 1). This gene cluster organization is well conserved in species producing a T2SS apparatus. An additional open reading frame (ORF), *gspO,* homologous to the prepilin peptidase required for processing of Type IV secretion system prepilin, is present downstream of the *gspN* gene but in the opposite orientation, indicating that *gspO* is probably transcribed independently of the rest of the T2SS genes. Interestingly, two ORFs, *cc0170* and *cc0171*, encoding a hypothetical protein and a TonB-dependent receptor, respectively, are found immediately upstream of the T2SS genes (Fig. 1). *cc0170* is conserved among a large number (122 Blastp hits) of bacteria living in a wild variety of environments such as *Shewanella* species living in ocean or *Bacteroides* species found in the gut. This high degree of conservation implies that *cc0170* probably plays an important role in bacterial survival in different environments. Sequence analysis reveals that *cc0170* is homologous to putative lipoproteins or secreted proteins, indicating that this gene likely encodes an extracellular protein. Interestingly, the tandem organization of *cc0170* and T2SS genes found in *C. crescentus* is also conserved in others species such as the plant pathogen *Xhanthomonas campestris* and the marine species *Sphingomonas alaskensis* and *Hyphomonas neptunium* in a similar genetic organization (Fig. 1), suggesting that *cc0170* encodes the first characterized T2SS substrate in *C. crescentus*.

### T2SS genes are expressed in poor and rich media

T2SS gene expression was assessed by measuring *gspE* and *gspL* mRNA levels in CB15N cells grown in rich culture medium (PYE). *gspE* and *gspL* encode a putative cytoplasmic ATPase and an inner membrane protein promoting secretion pore opening, respectively. *gspE* and *gspL* mRNA levels are represented as cycle threshold (CT) ratios calculated from qRT-PCR raw data (see Methods).

**Figure 2:**
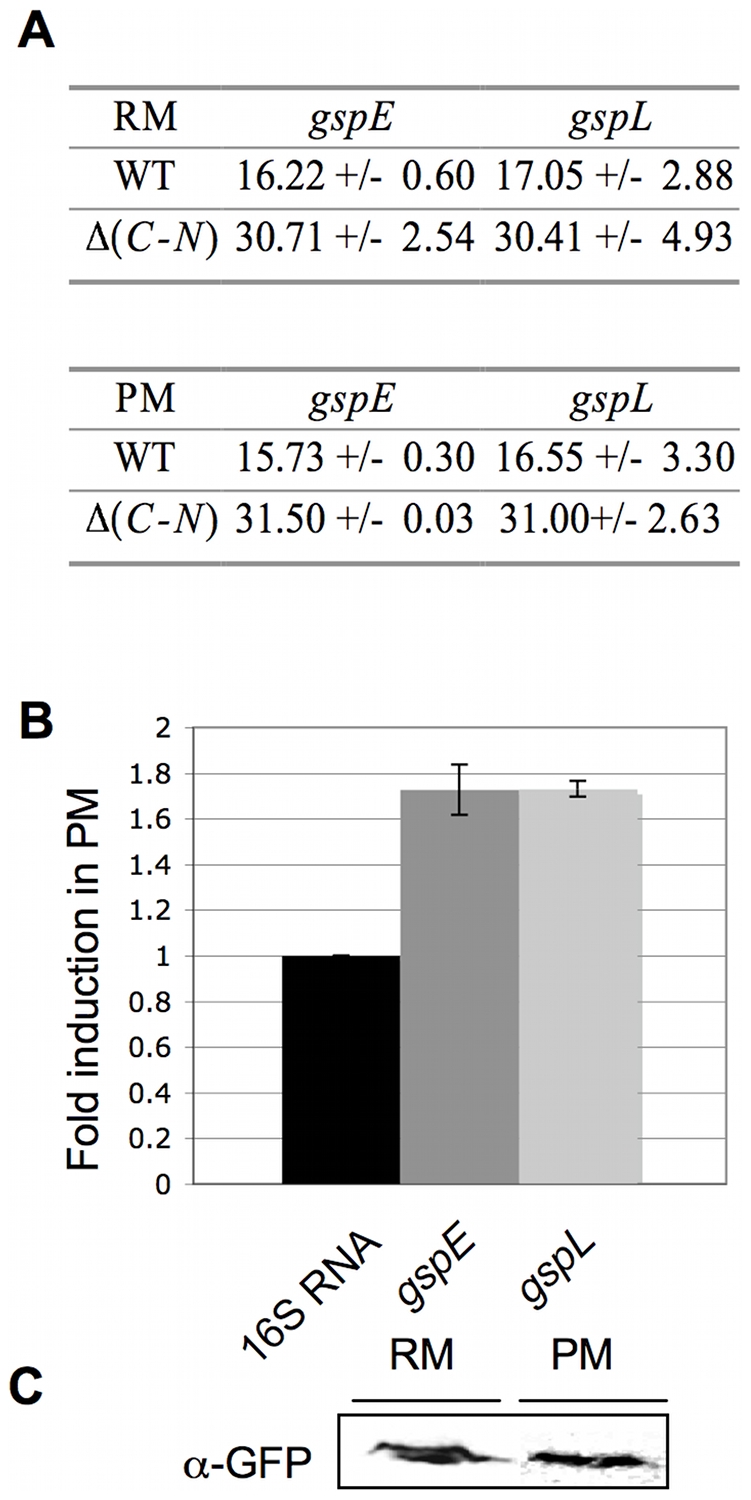
Expression of *gspL* and *gspE* in rich (RM) and poor (PM) media. (A) Cycle Threshold (CT) averages for *gspE* and *gspL* (see Methods). CT averages were determined by qRT-PCR performed on mRNA extract from CB15N (WT) and CB15N Δ(*gspC*-*gspN*) (Δ(*C-N*)) grown in poor (PM) and rich (RM) media. (B) Fold induction in PM (on the Y-axis) for *gspE* and *gspL.* For each target gene, the relative quantity of mRNA was calculated as the ratio of CT averages obtained in PM compared to RM. Each relative quantity was normalized by the 16S RNA induction in the two media. (C) Production of mGFP-GspL in PM and RM. Immunoblots were done on cellular fractions from CB15N *gspL*::*mgfp*-*gspL* grown in RM and PM with anti-GFP antibodies. Total proteins contained in cellular extracts used in (C) were detected by SDS-PAGE electrophoresis and silver nitrate staining as loading control for immunoblot.

Both *gspE* and *gspL* are expressed in rich medium, since their CT is significantly decreased in CB15N relative to CB15N Δ*gspC*-*N* in which the whole operon was knocked out (Fig. 2A). In order to generate more biologically relevant conditions, cells were grown in 1/5X PYE, hereafter referred to as poor medium. Under these conditions, *gspE* and *gspL* expression undergoes a 1.7 fold increase (Fig. 2B). To confirm these observations at the protein level, we constructed a strain carrying a *mgfp*-*gspL* fusion as the unique chromosomal copy and analyzed mGFP-GspL production by anti-GFP immunoblotting. mGFP-GspL is detected in rich and poor media at similar levels (Fig. 2C).

### T2SS triggers ElpS secretion in poor medium

**Figure 3:**
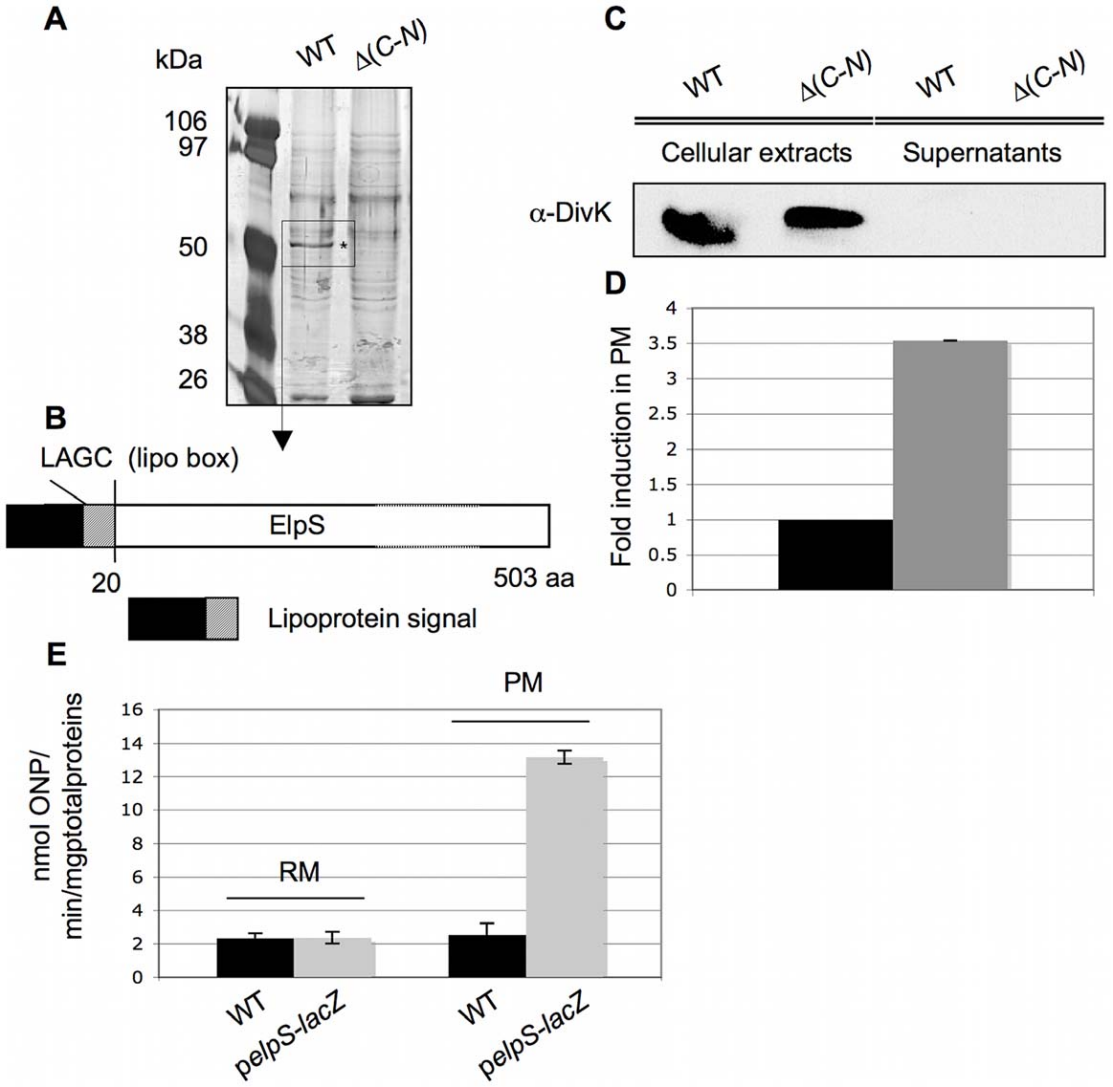
Release of ElpS depending on T2SS presence. (A) Extracellular proteins were detected by SDS-PAGE electrophoresis and silver nitrate staining on PRMM-precipitated supernatants from CB15N (WT) and CB15N Δ(*gspC*-*gspN*) (Δ(*C*-*N*)) grown in poor medium (PM). The indicated band (*) corresponds to ElpS identified by mass spectrometry. (B) ElpS predicted domains. *In silico* analysis of ElpS sequence indicates that the protein carries a predicted 20 amino acids lipoprotein signal containing a specific “lipo box” at position 17–20. (C) Absence of DivK in precipitated supernatant. Immunoblot with anti-DivK antibodies was done on cellular extracts and precipitated supernatant from CB15N (WT) and CB15N Δ(*gspC*-*gspN*) (Δ(*C*-*N*)) strains grown in PM. (D) Induction of *elpS* in PM. Cycle threshold averages for *elpS* were determined by RT PCR assays on RNA extract from CB15N grown in PM and RM. The fold induction in PM (on the Y-axis) of *elpS* was calculated as the ratio of CT averages in PM compared to RM and normalized by the 16SRNA relative quantity. (E) Activation of the *elpS* promoter in PM. β-galactosidase assays were performed on cellular extracts prepared under non-denaturing conditions from CB15N (WT) and CB15N/pSKoriT-kan-p*elpS*-*lacZ* grown in RM and PM.

The potential role of the T2SS was first determined by comparing protein secretion profiles between CB15N and CB15N Δ*gspC-N* in rich, poor and minimal media. Briefly, extracellular proteins were precipitated, separated on polyacrylamide gel and stained with silver nitrate. *C. crescentus* secretes a large amount of proteins in all experimental conditions but with different patterns (data not shown). Remarkably, one protein of about 50 kDa is strongly produced and secreted from CB15N cells grown in poor medium (Fig. 3A). The corresponding band is absent in CB15N Δ*gspC*-*N* culture supernatant, suggesting that its secretion relies on T2SS. This protein was identified by mass spectrometry as Cc0170, hereafter named ElpS for Extracellular lipoprotein produced under phosphate Starvation (Fig. 3B). As a lysis control, the cytoplasmic response regulator DivK was detected in cellular fractions but not in supernatants, indicating that secretion profiles indeed reflect protein secretion (Fig. 3C). Furthermore, qRT-PCR performed on CB15N grown in rich and poor media reveals a 3.5 fold upregulation of *elpS* in poor medium (Fig. 3D). The induction of *elpS* in poor medium was confirmed by a β-galactosidase assay performed on a strain carrying the *lacZ* reporter gene from *E. coli K12* under the control of the *elpS* promoter (Fig. 3E). Moreover, no significant β-galactosidase activity was found in CB15N grown in poor and rich media confirming that the observed β-galactosidase activity corresponds to a specific activation of the *elpS* promoter. *In silico* analysis of ElpS sequence (http://www.cbs.dtu.dk) highlights a conserved lipoprotein signal at the N-terminus (Fig. 3B). The lipoprotein signal was predicted using LipoP algorithm based on the Junker *et al*. method [22]. In particular, ElpS sequence is predicted to harbor a cleavage site for the Signal Peptidase II specific for lipoproteins maturation between aminoacid 19 and 20 with a score of 31.235 against a cutoff of 15.771. The authors also refers to a lipobox found at position -3 to +1 from the modified cystein with the following consensus sequence L-(A,S)-(G,A)-C. Accordingly, ElpS bears a lipobox at position 17–20 corresponding to a LAGC sequence. Nevertheless no other functional domains were found. Overexpression of *elpS*-*3flag* from the strong *lac* promoter on the low-copy pMR10 vector is toxic for cells lacking T2SS apparatus as evidenced by DivK release into the supernatant (data not shown), suggesting that a functional interaction may exist between ElpS and the T2SS apparatus.

**Figure 4:**
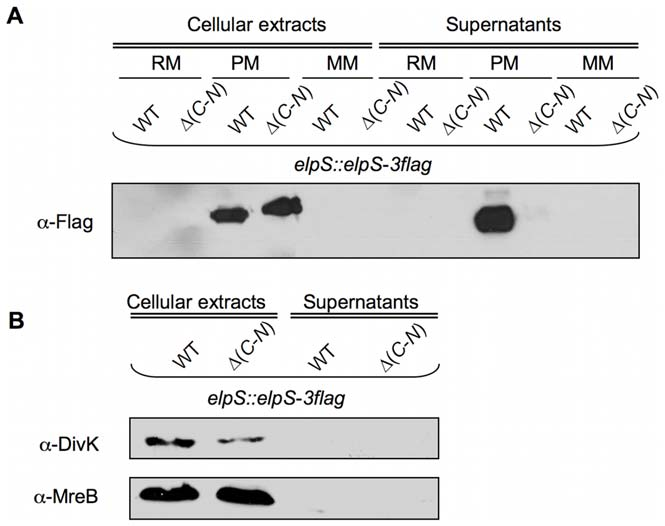
Production of ElpS-3Flag and mGFP-GspL in rich (RM), poor (PM) and minimal (MM) media. (A) Production of ElpS-3Flag in PM. Immunoblots with anti-Flag antibodies were done on cellular extracts and PRMM-precipitated supernatants from CB15N *elpS*::*elpS*-*3flag* (WT *elpS*::*elpS-3flag*) and CB15N Δ(*gspC*-*gspN*) *elpS*::*elpS*-*3flag* (Δ(*C*-*N*) *elpS*::*elpS*-*3flag*) grown in RM, PM and MM. Proteins contained in samples tested in (A) were detected by SDS-PAGE electrophoresis and silver nitrate staining as loading control for immunoblot. (B) Absence of detection of DivK and MreB in precipitated supernatants. Immunoblots with anti-DivK and anti-MreB antibodies were done on cellular extracts and PRMM-precipitated supernatants from CB15N *elpS*::*elpS*-*3flag* (WT *elpS*::*elpS-3flag*) and CB15N Δ(*gspC-gspN*) *elpS*::*elpS*-*3flag* (Δ(*C*-*N*) *elpS*::*elpS*-*3flag*) grown in PM.

Interestingly, ElpS-3Flag, expressed from the chromosome under its own promoter, is only produced in poor medium (Fig. 4A). No ElpS-3Flag could be detected in cellular extracts or supernatants from bacteria grown in rich or minimal media. This observation is in accordance with the transcriptional data previously presented showing that *elpS* is strongly upregulated in poor medium. ElpS-3flag accumulates in CB15N Δ*gspC*-*N* cells and is not released into the culture supernatant (Fig. 4A), indicating that the T2SS proteins are required for ElpS secretion. The absence of cytosolic DivK and MreB in the extracellular fraction from both cell lines rules out cell lysis (Fig. 4B).

Extracellular ElpS-3Flag is shorter in size than its cellular counterpart. This can be explained by a cleavage of the signal peptide by the Sec machinery during the export, which is generally observed with T2SS secreted proteins [23].

### ElpS is specifically produced and secreted under phosphate starvation

**Figure 5:**
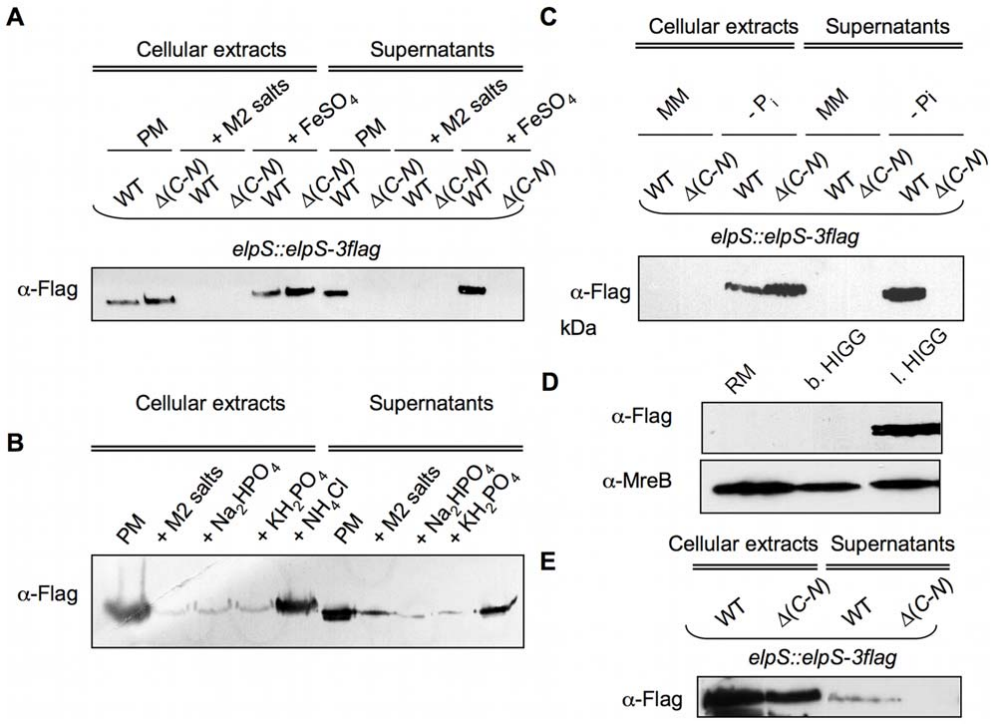
Production of ElpS-3Flag depending on phosphate concentration in culture media. (A) Inhibition of ElpS production by addition of M2 salts. Immunoblots with anti-Flag antibodies were done on cellular extracts and precipitated supernatants from CB15N *elpS*::*elpS*-*3flag* (WT *elpS*::*elpS-3flag*) and CB15N Δ(*gspC*-*gspN*) *elpS*::*elpS*-*3flag* (Δ(*C*-*N*) *elpS*::*elpS*-*3flag*) grown in poor medium (PM) supplemented with M2 salts or FeSO_4_. (B) Inhibition in ElpS production by addition of phosphate salts. Immunoblots with anti-Flag antibodies were done on cellular extracts and precipitated supernatants from CB15N *elpS*::*elpS*-*3flag* (WT *elpS*::*elpS-3flag*) grown in PM supplemented with Na_2_HPO_4_, KH_2_HPO_4_ or NH_4_Cl. (C) Production of ElpS in minimal medium (MM) depleted in phosphate salts (P_i_ salts). Immunoblots with anti-Flag antibodies were done on cellular extracts and precipitated supernatants from CB15N *elpS*::*elpS*-*3flag* (WT *elpS*::*elpS-3flag*) and CB15N Δ(*gspC*-gsp*N*) *elpS*::*elpS*-*3flag* (Δ(*C*-*N*) *elpS*::*elpS*-*3flag*) grown in minimal medium depleted or not in P_i_ salts. (D) Production of ElpS-3Flag in low phosphate medium. Immunoblots with anti-Flag and anti-MreB antibodies were done on cellular extracts from CB15N *elpS*::*elpS*-*3flag* grown in rich (RM), balanced phosphate HIGG (b.HIGG) and low phosphate HIGG (l. HIGG) media. (E) Production and secretion of ElpS-3Flag from CB15N *elpS*::*elpS*-*3flag* (WT *elpS*::*elpS-3flag*) and CB15N Δ(*gspC*-*gspN*) *elpS*::*elpS*-*3flag* (Δ(*C*-*N*) *elpS*::*elpS*-*3flag*) grown in low phosphate HIGG. Proteins contained in samples in A, B, C and E were detected by SDS-PAGE electrophoresis and silver nitrate staining as loading control for immunoblots.

*C. crescentus* does not produce any ElpS-3Flag when grown in minimal medium, which is surprising if we consider that minimal medium has lower nutrient content than poor medium. This observation implies that poor medium is devoid of a molecule repressing ElpS-3Flag synthesis, whereas this molecule is not limiting in minimal medium. The only nutrients more abundant in minimal medium than in poor medium are phosphate and ammonium salts (M2 salts), and FeSO_4_. Complementation of poor medium with M2 salts completely inhibited the production and the secretion of ElpS-3Flag, while 1 mM FeSO_4_ had no effect (Fig. 5A). Accordingly, *elpS* is not upregulated in iron-depleted medium (data not shown). In order to test which M2 salts component specifically represses ElpS synthesis, cells carrying *elpS*-*3flag* were grown in poor medium supplemented with phosphate or ammonium salts. The addition of NH_4_Cl had no effect on ElpS-3Flag level whereas phosphate salts (Na_2_HPO_4_ and KH_2_PO_4_) significantly reduced the production of ElpS-3Flag in both CB15N and CB15N Δ*gspC-N* strains (Fig. 5B). Accordingly, depletion of phosphate salts in minimal medium induces production of ElpS-3Flag in both strains (Fig. 5C). These results were confirmed using media containing a decrease range of phosphate concentrations. Briefly, ElpS-3Flag production is repressed in rich medium and in balanced phosphate HIGG but not in low phosphate HIGG medium (Fig. 5D). As expected, ElpS-3Flag is also secreted in low phosphate HIGG when T2SS is present (Fig. 5E). Taken together, these results demonstrate that production and secretion of ElpS are specifically upregulated under phosphate starvation.

### Intracellular ElpS is a membrane lipoprotein

**Figure 6:**
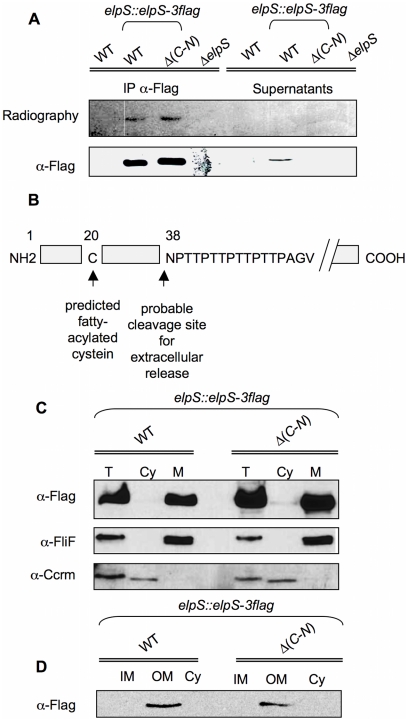
Radiolabeling with ^3^H-palmitic acid and subcellular localization of ElpS-3Flag. (A) ElpS-3Flag radiolabelled with ^3^H-palmitic acid was detected by autoradiography and immunoblots with anti-Flag antibody in cellular fractions after immunoprecipitation with mouse anti-Flag antibodies (IP a-Flag) and in 100-fold concentrated supernatants from CB15N (WT), CB15N *elpS::elpS-3flag* (WT *elpS*::*elpS*-*3flag*), CB15N Δ(*gspC-gspN*) *elpS::elpS-3flag* (Δ(*C-N*) *elpS::elpS-3flag*) and CB15N Δ*elpS* (Δ*elpS*) grown in poor medium containing 50 mCi/ml ^3^H-palmitic acid. (B) Probable cleavage site in ElpS sequence at position 38 from NH_2_-terminus based on N-terminal sequencing of the extracellular isoform of ElpS-3Flag. (C) Detection of ElpS-3Flag in membrane fraction. Immunoblots with anti-Flag, anti-FliF (membrane marker) and anti-CcrM (cytoplasm marker) were done on total lysates (T), soluble fractions (cytoplasm, Cy) and insoluble fractions (membrane, M) from CB15N *elpS::elpS-3flag* (WT *elpS*::*elpS*-*3flag*), CB15N Δ(*gspC-gspN*) *elpS::elpS-3flag* (Δ(*C-N*) *elpS::elpS-3flag*) grown in poor medium. (D) Detection of ElpS-3Flag in outer membrane fraction. Immunoblots with anti-Flag were done on inner membrane (IM), outer membrane (OM) and soluble fractions (cytoplasm, Cy) from CB15N *elpS::elpS-3flag* (WT *elpS*::*elpS*-*3flag*), CB15N Δ(*gspC-gspN*) *elpS::elpS-3flag* (Δ(*C-N*) *elpS::elpS-3flag*) grown in poor medium.

*In silico* analysis reveals a conserved lipoprotein sequence in the ElpS N-terminal domain. Precursors of lipoproteins are generally translocated as non-modified proteins through the inner membrane into the periplasm where lipidic groups are then added [24]. In order to verify the status of ElpS as a lipoprotein, CB15N, CB15N *elpS*::*elpS*-*3flag*, CB15N Δ*gspC*-*N elpS*::*elpS*-*3flag* and CB15N Δ*elpS* strains were grown in poor medium containing ^3^H-palmitic acid. Thereby, palmitoylated proteins will contain the isotope. Anti-Flag immunoprecipitates from cellular extracts or culture supernatants were subjected to SDS-PAGE and analysed by autoradiography. Cellular ElpS-3Flag appears to be palmitoylated irrespective of T2SS presence (Fig. 6A), whereas no signal is observed in the supernatant fraction. Accordingly, N-terminal sequencing performed on the secreted isoform of ElpS-3Flag indicates that the protein is cleaved at position 38 downstream the predicted fatty-acylated cysteine (Fig. 6B). These observations suggest a loss or a cleavage of the lipidic group when ElpS-3Flag is released in the extracellular medium. Lipoproteins are generally thought to be membrane-associated. Subcellular fractionation of CB15N *elpS*::*elpS*-*3flag* and CB15N Δ*gspC*-*N elpS*::*elpS*-*3flag* grown in poor medium confirmed the presence of ElpS-3Flag in the membrane fraction only, irrespective of T2SS presence (Fig. 6C). Moreover, additional fractionation assays showed that ElpS-3Flag is associated with the outer-membrane fraction and is never detected in the inner-membrane and cytoplasmic fractions (Fig. 6D).

### ElpS is a membrane surface-anchored protein subsequently released in the extracellular medium

**Figure 7:**
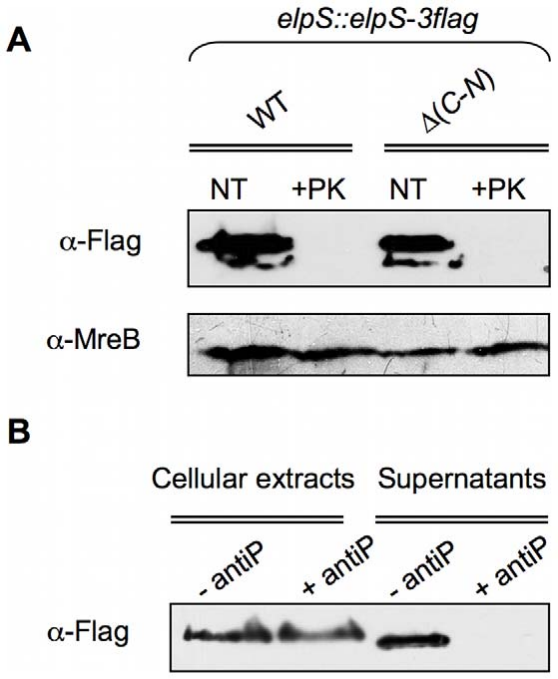
Membrane surface anchoring and protease-depending release of ElpS. (A) Sensitivity of ElpS-3Flag to proteinase K. Immunoblots with anti-Flag and anti-MreB antibodies were performed on total protein lysates harvested from cultures of CB15N *elpS::elpS-3flag* (WT *elpS*::*elpS*-*3flag*), CB15N Δ(*gspC-gspN*) *elpS::elpS-3flag* (Δ(*C-N*) *elpS::elpS-3flag*) grown in poor medium treated (+PK) or not (NT) with 2 µg/ml proteinase K. (B) Protease inhibitors sensitivity of ElpS-3Flag release in supernatant. Immunoblots with anti-Flag antibodies were performed on cellular extracts and precipitated supernatant from CB15N *elpS::elpS-3flag* grown in poor medium for 4.5 h then resuspended and incubated for 90 min in fresh medium containing (+ antiP) or not (-antiP) proteases inhibitors. Proteins contained in sample in (B) were detected by SDS-PAGE electrophoresis and silver nitrate staining as loading control for immunoblots.

To see whether ElpS is surface-exposed, ElpS-3Flag-producing CB15N *elpS*::*elpS*-*3flag* and CB15N Δ*gspC*-*N elpS*::*elpS*-*3flag* cells were treated with proteinase K to degrade membrane surface-exposed proteins. As shown in Fig. 7A, whereas the level of the cytoplasmic protein MreB remains stable, ElpS-3Flag signal entirely disappears in total extracts obtained from both proteinase K-treated cell lines, indicating that ElpS-3Flag is located at the external face of the outer membrane independently of T2SS. Similar treatment was performed on lysed cells without previous inactivation of proteinase K. In this case, proteinase K treatment led to a complete loss of cellular proteins including MreB (data not shown). Interestingly, treatment of CB15N *elpS*::*elpS*-*3flag* grown in poor medium with proteases inhibitors prevents ElpS release in the extracellular medium (Fig. 7B). Taken together, these data suggest that ElpS is independently sorted to bacterial surface and is subsequently cleaved by a T2SS-secreted protease and released in the environment.

### ElpS is associated with an alkaline phosphatase activity

**Figure 8:**
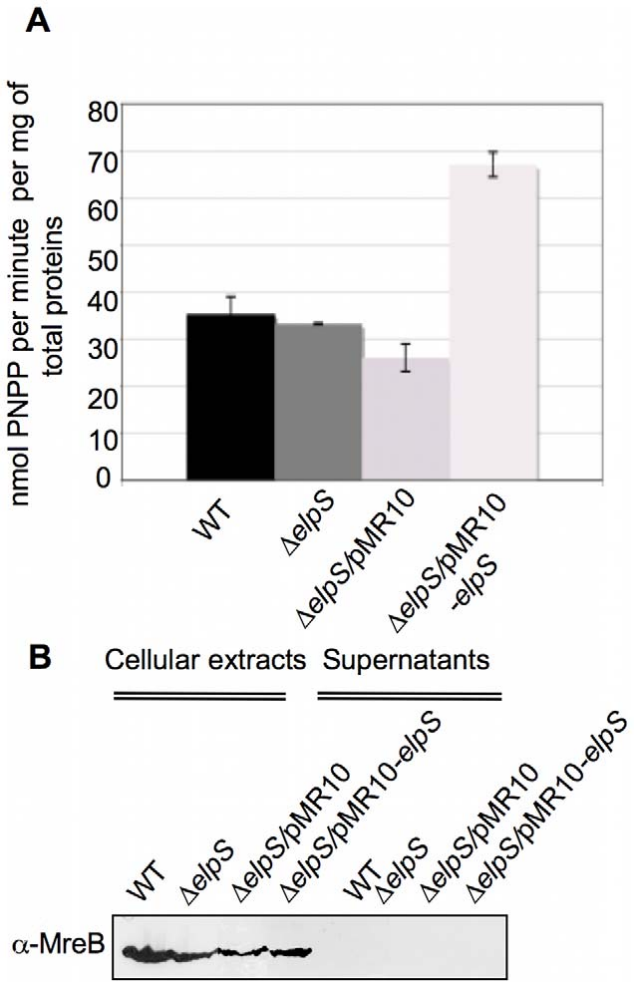
Increase of alkaline phosphatase activity in concentrated supernatants by ElpS overexpression. (A) Alkaline phosphatase assays on concentrated supernatants. Specific alkaline phosphatase activity was measured by degradation of 5 mg/ml *p*-nitrophenyl phosphate (PNPP) in 100-fold concentrated supernatants from CB15N (WT), CB15N Δ*elpS* (Δ*elpS*), CB15N Δ*elpS*/pMR10 (Δ*elpS*/pMR10) and CB15N Δ*elpS*/pMR10*-elpS* (Δ*elpS*/pMR10*-elpS*) grown in poor medium. (B) Absence of detection of MreB in concentrated supernatants. Immunoblot with anti-MreB antibodies was done on cellular extracts and 100-fold concentrated supernatants from strains tested in (A) grown in poor medium. Proteins contained in samples tested in (A) were detected by electrophoresis and silver nitrate staining as a loading control for immunoblot.

Since ElpS is specifically synthesized in response to phosphate starvation, it may promote release of P_i_ from organophosphate sources. Among the potential enzymatic activities responsible for P_i_ release from various substrates, alkaline phosphatase activity was measured in 100-fold concentrated culture supernatants using p-nitrophenyl phosphate as a substrate. The level of alkaline phosphatase activity remains unchanged in CB15N Δ*elpS.* In contrast, overexpression of *elpS* from pMR10 under the control of the *lac* promoter in CB15N Δ*elpS* leads to a significant increase of the alkaline phosphatase activity (Fig. 8A). These results suggest that other alkaline phosphatases masks or compensates a loss of phosphatase activity in the absence of ElpS synthesis. The absence of alkaline phosphatase activity in re-folded ElpS purified under denaturating conditions from *E. coli* and in extracellular immunoprecipitated ElpS-3Flag fractions from *C. crescentus* grown in poor medium suggests that ElpS is not an alkaline phosphatase itself but rather an activator of another still unknown phosphatase. The absence of the cytoplasmic MreB in the culture supernatants rules out any potential cell lysis that might explain the increase in alkaline phosphatase activity in the *elpS*- overexpressing cells supernatant (Fig. 8B).

## Discussion

In this paper, we demonstrate that phosphate starvation triggers production and T2SS-dependent release in the extracellular medium of a membrane-bound lipoprotein, ElpS. The constitutive production of a T2SS apparatus in *C. crescentus*, irrespective of environmental conditions, suggests that T2SS-dependent secretion of ElpS is controlled at another level, such as the transcriptional level since *elpS* expression is strongly induced under phosphate starvation. Accordingly, in *Aeromonas hydrophila* and *Vibrio cholerae*, constitutive expression of *exe* and *eps*, which encode T2SS components, is balanced by the regulated expression of genes encoding the cognate secreted product [21]. Similarly, the growth-phase-dependent secretion of AspA protease in *Aeromonas hydrophila* mainly relies on *aspA* transcriptional modulation [25]. Constitutive expression of a secretion apparatus combined with a tight regulation of secreted substrate production may offer an energetic advantage and a rapid adaptation to changing environments.

The abundance of lipoproteins in bacteria [22], [26], [27], [28] suggests the existence of many membrane-associated activities. Yet, the functions of most of them are still unknown. Lipoproteins are bound to membrane via a fatty-acyl group linked to a conserved cysteine and are sorted via the Lol system to the inner or outer membranes based on the identity of the amino acid following the cysteine [29], [30]. In particular, the presence of an aspartate following the conserved cysteine is considered as an inner membrane retention signal. Some atypical lipoproteins can also contains particular retention signals after the cystein such as CKVE/CGGG/CGDD/CQGS found in 5% of *P. aeruginosa* lipoproteins [31]. Other lipoproteins are conversely targeted to the outer membrane. In some cases, lipoproteins must also reach the external side of the outer membrane. Similarly to *Klebsiella oxytoca* pullulanase [9], ElpS is a membrane surface-anchored lipoprotein subsequently released in the environment. It is not always clear how lipoproteins are transported to the bacterial envelope [32], [33]. For instance, the export of pullulanase occurs through T2SS apparatus [34] whereas *Pseudomonas aeruginosa* HxcQ liposecretin is self-piloted to the outer membrane [35]. Unlike pullulanase, ElpS does not exhibit any known inner membrane retention signal and is consequently supposed to be addressed to the outer membrane. However, contrary to a non-secreted variant of pullulanase that accumulates in the periplasm [36], ElpS crosses the outer membrane in a T2SS-independent manner. Our results suggest that ElpS release in the environment is mediated by another T2SS-secreted protein. Indeed, secretion of ElpS in the environment is likely due to the proteolytic cleavage of ElpS by a T2SS-secreted protease. The absence of radiolabeling with ^3^H-palmitate of the extracellular ElpS isoform indicates that the exoprotein is deacylated when secreted, contrary to pullulanase [10]. Moreover, N-terminal sequencing of the secreted isoform of ElpS indicates that the protein is probably cleaved just before the asparagine 38 located downstream the fatty-acylated cysteine. Surprisingly, all detectable cellular ElpS is sensitive to proteinase K treatment, which led us to the idea that ElpS is primarily and transiently located in the outer membrane before secretion into the medium. Consistent with this hypothesis, enteropathogenic *E. coli* is able to accumulate an outer membrane-bound isoform of its major enterotoxin, collicin A, prior to release in its host cell [37]. Therefore, we can speculate that the release of ElpS relies on a specific signal such as an increase in population density, biofilm initiation or a drastic limiting threshold in inorganic phosphate concentration.

Phosphate is an important limiting nutrient in *C. crescentus* environment. *C. crescentus* is able to considerably increase stalk length when cells undergo phosphate starvation [16], thereby optimizing nutrient exchange [38]. Interestingly, stalk extracts harvested from *C. crescentus* grown in low phosphate HIGG medium contain many outer membrane proteins including Cc0171, a TonB-dependent receptor, and ElpS [39]. This particular protein content implies that the stalk must be an essential organelle for nutrient uptake and suggests that ElpS and Cc0171 play a role in the adaptation to nutrient-deficient media. The co-occurrence of *elpS* and *cc0171* homologs in genomes probably reflects their functional interaction. Interestingly, RT PCR analysis show that, contrary to *elpS*, *cc0171* is constitutively induced in rich and poor media suggesting that both genes are independently regulated (data not shown). TonB-dependent receptors are generally sensors of extracellular signals [40], and Cc0171 could be involved in the detection of phosphate starvation and the establishment of an adaptive response through the induction of *elpS*. Nevertheless, deletion of *cc0171* does not suppress the production of ElpS-3Flag in poor medium (data not shown). We also assume that Cc0171 does not act on *elpS* induction under phosphate starvation. On the contrary, the TonB-dependent receptor may be involved in phosphate uptake through the activation of ElpS. However, no significant change in intracellular phosphate concentration is detected when cells are switched from rich to poor medium which could be due to the low sensitivity of the method used (data not shown). ElpS is specifically produced under phosphate starvation and is associated with an increased secreted alkaline phosphatase activity when overproduced, Together, our data suggest that ElpS participates in phosphate mobilization.

## Materials and Methods

### Strains, plasmids and growth conditions

All strains of *E. coli* and *C. crescentus* were derivatives of DH10B and CB15N strains, respectively. DNA manipulations were performed according to standard techniques. Restriction enzymes were purchased from Roche, and primers were purchased from Eurogentec. Strains and plasmids are summarized in Table S1 [47] and their mode of construction is described in the Text S1. *E. coli* strains were grown in Luria Bertani broth medium (LB broth, Difco) supplemented with 100 µg/ml ampicillin or 50 µg/ml kanamycin when necessary. *C. crescentus* strains were grown in Peptone Yeast extract (PYE) [41], 1/5X PYE (5-fold diluted PYE except 1 mM MgSO_4_ and 0.5 mM CaCl_2_+0.2% glucose), M2G [42], in balanced phosphate (200 µM) or low phosphate (30 µM) Hutner base-imidazole-buffered-glucose-glutamate HIGG at 30°C [43].

### RNA preparation

Total RNAs were extracted from CB15N, CB15N Δ*gspC*-*N* and CB15N Δ*elpS* (all cultured in duplicate) as follows: 45 ml culture (OD_660 nm_ = 0.5 at 30°C) were centrifuged for 15 min at 3500 rpm. Bacterial pellets were resuspended in 100 µl 10% SDS and 20 µl 20 mg/ml proteinase K and incubated for 1 h at 37°C with shaking. Five ml of TRIzol® Reagent (Invitrogen) were added and suspensions were vigorously shaken. After a 10 min incubation at 65°C, 1 ml chloroform was added to the suspensions and the mixtures were shaken and incubated for 5–10 min at room temperature. Samples were centrifuged for 15 min at 14,000 rpm at 4°C, 2.5 ml propan-2-ol were added to the aqueous phases and samples were stored overnight at -20°C. After centrifugation for 30 min at 14,000 rpm at 4°C, pellets were washed with 75% RNase-free ethanol. Supernatants were discarded and pellets were dried for 15 min at room temperature. Total RNA samples were resuspended in 100 µl RNase-free H_2_O, incubated for 10 min at 55°C and stored at −80°C. The integrity of the RNA and the absence of DNA were checked by gel electrophoresis. RNA was quantified with a NanoDrop (ND-1000, Thermo Fisher Scientific).

### Quantitative real-time RT-PCR

RNA samples were obtained using DNA-free kit (Ambion) and reverse-transcription was performed with SuperScript II Reverse Transcriptase (Invitrogen). Primers listed in Table S2 were designed with PrimerExpress® 2.0 (Applied Biosystems). PCR products ranged from 80 to 100 bp. Real-time PCR reactions were performed with SYBR Green Mix (Applied Biosystems) in an Applied Biosystems Step One Plus real-time PCR instrument. Ratios were calculated using the CT method for each primer. CT (Cycle Threshold) was defined as the number of cycles required for the fluorescent signal to cross a defined threshold of signal (i.e., up to background level). CT levels were thus inversely proportional to the amount of target nucleic acid in the sample. CTs <29 are strong positive reactions indicative of abundant target nucleic acid in the sample. CTs of 30–40 are weak reactions indicative of minimal amounts of target nucleic acid. To determine relative expression levels CTs obtained in 1/5X PYE and in PYE were normalized to 16S RNA, averaged and compared.

### β-galactosidase assays

*C. crescentus* strains were grown in PYE and 1/5X PYE media for 6 h at 30°C. Cells were harvested by centrifugation for 10 min at 6000 rpm and resuspended in 1/2 volume of Z-Buffer (16.1 g/l Na_2_HPO_4_.7H_2_O; 5.5 g/l NaH_2_PO_4_.7H_2_O; 0.75 g/l KCl; 0.246 g/l MgSO_4_.7H_2_O; 0.0005% NP40) and 1/185 volume of β-mercaptoethanol. Cell lysis was obtained by adding 1/10 volume of glass beads and vortexing 6 cycles of 30 seconds spaced with 30 seconds cycles on ice. Membrane and beads were separated from cytoplasmic fractions by centrifugation for 10 min at 6000 rpm. Supernatants containing soluble proteins were collected, mixed with 1/5 volume of 4 mg/ml ortho-Nitrophenyl-β-galactoside (ONPG) and incubated for 1 min to 1 h at room temperature. ONPG degradation was monitored at OD_420 nm_ by formation of an yellow product ortho-Nitrophenyl (ONP) and stopped by the addition of 0.3 M Na_2_CO_3_. Specific β-galactosidase activity was determined in nmol of degraded ONPG (ε_420 nm_ = 4500 M^−1^) per min per mg of total protein. Total protein concentrations were determined using Bradford assay (Bio-Rad Protein Assay).

### Preparation of cellular extracts and precipitated supernatants under denaturing conditions

*C. crescentus* was grown in PYE, 1/5X PYE, M2G, balanced or low phosphate HIGG to OD_660 nm_ = 0.5 at 30°C. Culture supernatants were isolated by a 10 min centrifugation at 3500 rpm. Corresponding supernatants were filtered on 0.22 µM filters and proteins were precipitated using a pyrogallol red-molybdate-methanol (PRMM) protocol <Caldwell, 2004 #5>. Protein pellets were resuspended in 1/150 volume of SDS-PAGE loading buffer (1 M Tris-HCl pH 6.8; 2% SDS; 10% glycerol; 16% b-mercaptoethanol; 0.06% bromophenol blue). Cellular extracts were obtained by resupending bacterial pellets in SDS-PAGE loading buffer to a final corresponding OD_660 nm_ = 2. All protein samples were boiled for 5 min and stored at −20°C.

### Preparation of cellular extracts and supernatants under non-denaturing conditions

*C. crescentus* was grown in 1/5X PYE to OD_660 nm_ = 0.5 at 30°C except when notified. Culture supernatants were 100 fold-concentrated on Amicon (Amicon® Ultra-10k Millipore). Cellular extracts were obtained by resuspending bacterial pellets in PBS (140 mM NaCl; 2 mM KCl; 10 mM Na_2_HPO_4_; 2 mM KH_2_PO_4_) to a final OD_660 nm_ = 2 and sonicating 6 times for 30 s. Protein samples were stored at −20°C.

### Silver nitrate staining and immunoblots of protein extracts

When prepared under non-denaturing conditions, samples were first mixed with 2X SDS-PAGE loading buffer and boiled for 5 min. Proteins were then separated on 12% polyacrylamide gels and detected by silver nitrate staining or transferred to a PVDF membrane for immunoblotting. For silver nitrate staining, gels were first fixed for 20 min in 50% methanol, 5% acetic acid, then washed for 10 min in 50% methanol and for 2 h in H_2_O. Gels were incubated in sensitizing solution containing 0.02% sodium thiosulfate and washed again twice for 1 min in H_2_O. Proteins were revealed by incubating the gels another 20 min in 0.1% silver nitrate followed by 2 washes for 1 min in H_2_O and one last incubation in 0.04% formalin, 2% sodium carbonate. The staining reaction was stopped in 5% acetic acid. ElpS-3Flag, DivK, MreB, mGFP-GspL, FliF and CcrM were immunodetected with 1∶1000 anti-Flag (Monoclonal anti-Flag® M2 or rabbit anti-Flag® antibodies, Sigma-Aldrich), 1∶5000 anti-DivK [44], 1∶5000 anti-MreB [45], 1∶1000 anti-GFP (Monoclonal Anti-Green Fluorescent Protein (GFP) (Sigma-Aldrich), 1∶2000 anti-FliF and 1∶2000 anti-CcrM antibodies, respectively.

### Protein radiolabeling with ^3^H-palmitic acid

PYE precultures at OD_660 nm_ = 0.1 were diluted in 1/5X PYE containing 50 mCi/ml ^3^H-palmitic acid and grown for 6 h at 30°C. Cellular extracts and supernatants were prepared under non-denaturing conditions as above. ElpS-3Flag was immunoprecipitated with 1∶1000 anti-Flag antibodies (Monoclonal anti-Flag® M2, Sigma-Aldrich) and Protein A - Sepharose® 4B (Invitrogen). Immunoprecipitates were resuspended in 100 µl SDS-PAGE loading buffer and concentrated supernatants were mixed with 2X SDS-PAGE loading buffer. Proteins were boiled for 5 min and 10 µl of each sample were separated on 12% polyacrylamide gels. Radioactive signal was enhanced using fluorography [46]. ElpS-3Flag was immunodetected with rabbit anti-Flag antibodies.

### N-terminal sequencing

Proteins extracts were obtained from 10000-fold concentrated supernatants on Amicon from CB15N *elpS*::*elpS*-*3flag* and CB15N Δ*elpS* grown for 6 h in 1/5X PYE, separated on 12% polyacrylamide gel and transferred to PVDF membrane. Proteins were detected by Coomassie blue staining using the Pierce Coomassie Plus Protein Assay Reagent and Kit (Thermo Scientific) and ElpS-3Flag was immunodetected with anti-Flag antibodies. The band corresponding to ElpS-3Flag was cut through the simultaneous analysis of total proteins profiles and immunoblot. The N-terminal end sequence of ElpS was obtained from Pick-n-Post (http://www.pick-n-post.com).

### Cell fractionation assays

Cells (400 ml) were grown to OD_660 nm_ = 0.5 in 1/5 X PYE at 30°C. Cells were harvested by centrifugation at 4°C, washed in 50 ml CBB buffer (20 mM Tris-Cl pH 8.0, 25 mM NaCl, 5 mM EDTA, 3.6 mM β-mercaptoethanol), and resuspended in 10 ml CBB buffer with a protease inhibitor cocktail (Complete EDTA free, Roche). Cells were passed twice through a French pressure cell (∼20–25,000 psi) and the lysate was cleared by centrifugation at 20,000× *g* for 15 min at 4°C. The cleared lysate was then centrifuged at 150,000× *g* for 2 h at 4°C, and a sample of the supernatant was taken for analysis. The pellet was resuspended in 1 ml CBB buffer and spun at 105,000× *g* for 1 h. The supernatant was discarded and the washed pellet was resuspended in 1 ml CBB with 0.5% SDS and used for analysis. The protein content of the cleared lysate was measured using a Bradford assay and was used to normalize load volumes between samples. Within a fractionation sample, equivalent volumes were loaded on gels. Samples were mixed with 5X SDS-PAGE loading buffer, boiled for 5 min, separated on 12% polyacrylamide gels, transferred to PVDF membranes, and blotted with anti-Flag, anti-FliF and anti-CcrM antibodies.

### Inner and outer membranes preparation

Cells (300 ml) were grown to OD_660 nm_ = 0.5 in 1/5 X PYE at 30°C. Cells were harvested by centrifugation at 4°C, washed 3 times in 50 ml 50 mM ammonium bicarbonate (AmBic) pH 8 and resuspended in 5 ml AmBic. Cells were sonicated at maximal intensity 20 times for 5 s and kept on ice between each cycle. The lysate was cleared by centrifugation for 10 min at 12,000× *g* at 4°C. The cleared lysate was then centrifuged for 40 min at 100,000× *g* at 4°C, and a sample of the supernatant was taken for analysis (cytoplasmic fraction). The pellet was resuspended in 1 ml 1% sodium lauryl sarkonisate and spun for 40 min at 100,000× *g*. The supernatant corresponding to solubilized inner membrane proteins was taken for analysis and the pellet was resuspended in 1 ml 2.5 M NaBr and incubated for 30 min at 4°C and then ultracentrifuged for 40 min at 100,000× *g*. The outer membrane pellet was resuspended in 1 ml 100 mM Na_2_CO_3_ and spun again for 40 min at 100,000 g ×. The OM proteins was resuspended in SDS-PAGE loading buffer. Within a fractionation sample, equivalent volumes were loaded on gels. Samples were mixed with 2X SDS-PAGE loading buffer, boiled for 5 min, separated on 12% polyacrylamide gels, transferred to nitrocellulose membrane, and blotted with anti-Flag antibodies.

### Proteinase K sensitivity assays

Cells were grown to OD_660 nm_ = 0.5 in 1/5X PYE at 30°C. Cultures were treated or not with 2 µg/ml proteinase K for 30 min at 37°C. Cells were harvested by centrifugation for 10 min at 3500 rmp, the supernatant was discarded and the pellet washed in 1 volume of M2 salts (10 mM NH_4_Cl, 6 mM Na_2_HPO_4_ and 4 mM KH_2_PO_4_). Cells were finally resuspended in 1/4 volume of M2 salt containing 10 mM phenylmethanesulphonylfluoride (PMSF). Samples were mixed with 2X SDS-PAGE loading buffer, separated on a 12% polyacrylamide gel and transferred to nitrocellulose membrane. ElpS-3Flag and MreB were immunodetected using anti-Flag and anti-MreB antibodies respectively.

### Anti-proteases treatment on cells producing ElpS-3Flag

CB15N *elpS*::*elpS-3flag* was grown in 1/5X PYE for 4.5 h at 30°C. Cells were then harvested by centrifugation for 10 min at 3500 rpm and resuspended in an equal volume of fresh 1/5X PYE containing or not a cocktail of protease inhibitors (Complete, EDTA-free; Roche) and incubated for 90 min at 30°C. ElpS-3Flag was immunodetected using anti-Flag antibodies in cellular extracts and precipitated supernatant from resulting samples.

### Alkaline phosphatase assays on concentrated supernatants

Alkaline phosphatase assays were performed on 100 fold-concentrated supernatants obtained under non-denaturating conditions from cells grown for 4 h in 1/5X PYE. Specific alkaline phosphatase activity was measured by degradation of 1 mg/ml p-Nitrophenyl Phosphate (PNPP) in 100 mM pH 7.9 Tris-HCl, 2 mM MgCl2. P-Nitrophenol formation induced by PNPP cleavage was monitored at OD420 nm. Alkaline phosphatase activity was calculated in nmol of degradated PNPP (e420 nm = 18000 M-1)/min/mg of total protein. Total protein concentrations were determined using micro-Bradford assays (Bio-Rad Protein Assay).

## Supporting Information

Table S1 Strains and plasmids.
(0.05 MB DOC)

Table S2 Primers used for RT PCR assays.
(0.03 MB DOC)

Text S1 Strain and plasmid constructions.
(0.04 MB DOC)
