# Supplementary material for: Phosphate Starvation Triggers Production and Secretion of an Extracellular Lipoprotein in Caulobacter crescentus
Source: PLoS One. 2010 Dec 2;5(12):e14198. doi: 10.1371/journal.pone.0014198 (PMC2996285; doi:10.1371/journal.pone.0014198)
Supplement: Table S1 — Strains and plasmids. (0.05 MB DOC) [file pone.0014198.s001.doc]

| **Strains and plasmid** | **Description** | **References** |
| --- | --- | --- |
| Strains |  |  |
| CB15N | *C. crescentus* CB15N (wild type) | [47] |
| CB15N Δ(*gspC*-*gspN)* | CB15N deletant strain for *gspC* to *gspN* genes (*cc0173* to *cc0184*) | This study |
| CB15N *gspL*::*mgfp*-*gspL* | CB15N carrying on the chromosome as the sole copy of *gspL*, a translational fusion of *gspL* with *mgfp* (coding for mutated GFP A206K to prevent dimerization) at the 3’end terminal domain | This study |
| CB15N*/*pMR10*-elpS-3flag* | CB15N carrying the translational fusion *elpS*-3flag on the replicative vector pMR10 under control of the *lac* promoter | This study |
| CB15N Δ(*gspC*-*gspN*)*/*pMR10*-elpS-3flag* | CB15N Δ(*gspC*-*gspN*) carrying the translational fusion *elpS*-3*flag* on the replicative vector pMR10 under control of the *lac* promoter | This study |
| CB15N *elpS*::*elpS*-*3flag* | CB15N carrying on the chromosome as sole copy of *elpS*, a translational fusion of *elpS* with *3flag* at the C-terminal domain | This study |
| CB15N Δ(*gspC*-*gspN*) *elpS*::*elpS*-*3flag* | CB15N Δ(*gspC*-*gspN*) carrying on the chromosome as sole copy of *elpS*, a translational fusion of *elpS* with *3flag* at the C-terminal domain | This study |
| CB15N Δ*elpS* | CB15N deletant strain for *elpS* gene | This study |
| CB15N Δ*elpS*/pMR10-empty | CB15N deletant strain for *elpS* gene carrying the empty replicative vector pMR10 | This study |
| CB15N Δ*elpS*/pMR10-*elpS* | CB15N deletant strain for *elpS* gene carrying a copy of *elpS* on the pMR10 under control of the *lac* promoter | This study |
| CB15N/pSKoriT-*kan*-p*elpS*-*lacZ* | CB15N expressing *lacZ* under control of the *elpS* promoter on the integrative vector pSKoriT-*kan* | This study |
| Plasmids |  |  |
| pMR10 | Low copy number replicative cloning vector in *E.coli* and *C. crescentus* | (Mohr *et a*l., unpublished) |
| pMR10-*elpS* | Low copy number replicative in *E. coli* and *C. crescentus* carrying a copy of *elpS* under control of the *lac* promoter | This study |
| pMR10-*elpS-3flag* | Replicative vector in *E. coli* and *C. crescentus* carrying a copy of the translational fusion *elpS*-*3flag* under control of the *lac* promoter | This study |
| pSKoriT-*kan*-p*elpS*-*lacZ* | Integrative vector in *C. crescentus* carrying a fusion of the *elpS* promoter with the *lacZ* reporter gene from *E. coli* K12 | This study |
| pNPTS138-upstream-downstream-*gspC-N* | Integrative vector in *C. crescentus* carrying 500 bp 5’ of *gspC* adjacent to 500bp 3’ of *gspN*. | This study |
| pNPTS138-upstream-downstream-*elpS* | Integrative vector in *C. crescentus* carrying fused flanking regions (500 bp) of *elpS* | This study |
| pNPTS138-upstream-*mgfp*-*gspL* | Integrative vector in *C*. *crescentus* carrying a copy of *mgfp*-*gspL* fused to the the upstream region of *gspL* on the chromosome (500 bp) | This study |
| pNPTS138-*elpS*-*3flag*-downstream | Integrative vector in *C*. *crescentus* carrying a copy of *elpS-3flag* fused to the the downstream region of *elpS* on the chromosome (500 bp) | This study |
