# Supplementary material for: Phosphate Starvation Triggers Production and Secretion of an Extracellular Lipoprotein in Caulobacter crescentus
Source: PLoS One. 2010 Dec 2;5(12):e14198. doi: 10.1371/journal.pone.0014198 (PMC2996285; doi:10.1371/journal.pone.0014198)
Supplement: Table S2 — Primers used for RT PCR assays. (0.03 MB DOC) [file pone.0014198.s002.doc]

| Primer | Sequence |
| --- | --- |
| ELPSF | CGTTCGTGATGAGCTGTAATG |
| ELPSR | AACCAAACTGAGCCGCC |
| CC0171F | CTGGGCTTTGGCGAGAA |
| CC0171R | GCCCATGGCCAGACG |
| GspEF | TGAACGACAACAGCCGC |
| GSPER | CCTTGGTGTTGACCTGGGT |
| GSPLF | AATGACCTTGCTCAGCCTTC |
| GSPLR | TAACCGACAGGCCATAGTCCT |
