# Supplementary material for: Phosphate Starvation Triggers Production and Secretion of an Extracellular Lipoprotein in Caulobacter crescentus
Source: PLoS One. 2010 Dec 2;5(12):e14198. doi: 10.1371/journal.pone.0014198 (PMC2996285; doi:10.1371/journal.pone.0014198)
Supplement: Text S1 — Strain and plasmid constructions. (0.04 MB DOC) [file pone.0014198.s003.doc]

**Plasmids and strains construction**

For pMR10-*elpS* construction, the wild-type *elpS* allele and the downstream region of *elpS* (500 bp) were amplified with primers ELPSF-XhoI/ELPS-DOWNSTREAMR-EcoRI : AGTGCCCATAACGGCTCGAGGTGAAGCTGTATAGAAACCTAA / GAATTCACACGCTTCAGCGGCTCAGG. The resulting PCR products were first cloned into pGEM-Teasy (pGEM®-T Easy Vector System I Promega Ref A1360) and checked by DNA sequencing with primers UP-PGEMT/DO-PGEMT/ELPSF-XhoI/ELPS-DOWNSTREAMR-EcoRI: GTTTTCCCAGTCACGACG/ GGAAACAGCTATGACCA*.* EcoRI restricted inserts excised from recombinant pGEM-Teasy were cloned into pMR10. pMR10-*elpS* or empty pMR10 were introduced in CB15N or CB15N Δ*elpS* strains by electroporation as described by Gilchrist and Smit (1991). Positives clones for transformation were selected on PYE added with kanamycin and checked by colony PCR with primers PMR15F/PMR15R: TCAATGATGACCTGGTGCAT / TTGGGTAACGCCAGGGTTTT.

For pMR10-*elpS-3flag* construction, the fusion *elpS*-*3flag* was amplified with primers ELPSF-XhoI and ELPS-FLAGR-EcoRI : ACTGGACCGAATTCTTACTTGTCATCGTCATCCTTGTAATCGATGTCATGATCTTTATAATCACCGTCATGGTCTTTGTAGTCGTTGGTCGGCAGCGACGTGCAGGCGGT and first cloned into pGEM-Teasy. Cloned sequence was checked by DNA sequencing with primers UP-PGEMT/DO-PGEMT/ELPSF-*Xho*I*/*ELPS-FLAGR-*EcoR*I. XhoI/EcoRI restricted inserts excised from recombinant pGEM-Teasy were cloned into pMR10. pMR10-*elpS*-*3flag* was introduced in CB15N or CB15N Δ*gspC-N* strains by electroporation.

For pSKoriT*kan*-p*elpS*-*lacZ* construction, KpnI/BamHI restricted *lacZ* gene was excised from pCH110 and cloned into pSKoriT*kan* integrative vector. The *elpS* promoter (p*elpS*) was first amplified with primers PELPSF-KpnI/PELPSR-KpnI: **GGTACC**CACGATGAGGCTTTAGGCGA / **GGTACC**AACACAGGAGCCCCTCTGGTTT. Resulting PCR products were first cloned into pGEM-Teasy and checked by DNA sequencing with primers UP-PGEMT/DO-PGEMT: GTTTTCCCAGTCACGA / GGAAACAGCTATGACCATG. The p*elpS* was excised from the recombinant pGEM-Teasy with the restriction enzyme KpnI and cloned into pSKoriT*kan*-*lacZ*. Resulting pSKoriT*kan*-p*elpS*-*lacZ* was introduced in CB15N strain by electroporation. Positives clones for transformation were selected on PYE supplemented with kanamycin and checked by colony PCR with primers PELPSCHECKF/ILACZ: GGCCGACAAGAAGATCGTCT / GTTGGGTAACGCCAGGGTT.

Gene deletions or allelic replacements were obtained by two successive homologous recombinations between the *C. crescentus* genome and recombinant pNPTS138 (non replicative vector in *C. crescentus*). All recombinant pNPTS138 contain upstream and downstream regions (500 bp each) of the target gene to delete or to replace. For gene deletion, pNPTS138 only contains the upstream and downstream flanking regions fused to each other. For allelic replacement, pNPTS138 harbours modified target gene flanked with upsteam and downstream regions. A first recombination step occured between on of the common regions of the vector and the *C. crescentus* chromosome and was selected on kanamycin. A second recombination in the absence of selection pressure allowed the excision of the plasmid and led to the excision of the plasmid and in some cases to gene deletion or allelic replacement of the target gene. Positive clones for excision were selected on sucrose PYE (sensitivity to sucrose encoded by pNPTS138). Positive clones for gene deletion or allelic replacement were checked by colony PCR of *C. crescentus* chromosome and DNA sequencing.

Strain deleted for T2SS genes was obtained by recombination between pNPTS138-*upstream*-*downstream*-*gspC*-*N* and CB15N strain chromosome. For pNPTS138-*upstream*-*downstream*-*gspC*-*N* construction, upstream and downstream regions (500 bp each) of T2SS genes were separately amplified with primers T2SS-UPSTREAMF / T2SS-UPSTREAMR : CTGCAGCACGATGAGGCTTTAGGCGA/CTCGAGAGGAGCCCCTCTGGTTTCTC and T2SS-DOWNSTREAMF / T2SS-DOWNSTREAMR : CTCGAGGACGACGCGACTTCGTCGAA / GAATTCACACGCTTCAGCGGCTCAGG. Resulting PCR products were first cloned into pGEM-Teasy and checked by DNA sequencing with primers UP-PGEMT/DO-PGEMT. EcoRI/NcoI and NcoI/PstI-restricted upstream and downstream regions excised from recombinant pGEM-Teasy were simultaneously cloned into pNPST138 at EcoRI/PstI sites. Resulting pNPTS138-*upstream*-*downstream*-*gspC*-*N* was introduced in CB15N by electroporation. Clones deleted for *gspC*-*N* genes were checked by colony PCR with primers ΔT2SS-CHECKF / ΔT2SS-CHECKR : CTGGGTCTTGAGGACACCGAG / GTCAGCTTTCTTGTGCTGC.

Deletant strain for *elpS* gene was obtained by recombination between pNPTS138-*upstream*-*downstream*-*elpS* and CB15N chromosome. For pNPTS138-*upstream*-*downstream*-*elpS* construction, upstream and downstream regions (500 bp each) of *elpS* genes were separately and respectively amplified with primers ELPS-UPSTREAMF-PstI / ELPS-UPSTREAMR: CTGCAGCACGATGAGGCTTTAGGCGA / TTCGACGAAGTCGCGTCGTCAGGAGCCCCTCTGGTTTCTC and ELPS-DOWNSTREAMF / ELPS-DOWNSTREAMR-EcoRI: GAGAAACCAGAGGGGCTCCTGACGACGCGACTTCGTCGAA / GAATTCACACGCTTCAGCGGCTCAGG. Resulting PCR products were mixed and used as a matrix for a second PCR with primers ELPS-UPSTREAMF-PstI / ELPS-DOWNSTREAMR-EcoRI to obtain fusion products of flanking regions. Fused flanking regions were first cloned into pGEM-Teasy and checked by DNA sequencing with primers UP-PGEMT/DO-PGEMT. PstI/EcoRI restricted fragments obtained from recombinant pGEM-Teasy were cloned into pNPTS138. Resulting pNPTS138-*upstream*-*downstream*-*elpS* was introduced in CB15N by electroporation. Deletant clones for *elpS* were checked by colony PCR with primers ΔELPS-CHECKF / ΔELPS-CHECKR: CTGGGTCTTGAGGACACCGAG / GTCAGCTTTCTTGTGCTGC.

Allelic replacement of *gspL* by *mgfp*-*gspL* was obtained by homologous recombination between CB15N and pNPTS138-*upstream*-*mgfp*-*gspL*. Upstream region of *gspL* (500 bp) was amplified with primers GSPL-UPSTREAMF-PstI / GSPL-UPSTREAMR-BamHI : CTGATAACTGCAGGACAGCAACGACGTGGGCCG / CGGGGATCCACGTCGCGCAGCCAGGATCA and cloned into pGEM-Teasy. Insert was checked by DNA sequencing using UP-PGEMT/DO-PGEMT. PstI/BamHI restricted upstream region was cloned in pNPTS138 generating the pNPTS138-*upstream*-*gspL*. Sequence coding for mGFP and GspL were respectively amplified with primers GFPF-BamHI / GFPR-XbaI : GACCTAGGACAAGGCATTGACAGGATCCATGGTGAGCAAGGGCGA / TACCGATCCAGTACATCTAGACTTGTACAGCTCGTCCATG and GspLF-XbaI/ GspLR- EcoRI : AATGCCATGAGATCTAGATTGGACCCCCGAAGAATG/ATCCATTCAGAGAATTCTCATGGCTTTCTCCTGATG and cloned into pGEM-Teasy. Inserts were checked by DNA sequencing with primers UP-PGEMT/DO-PGEMT. *BamH*I/*Xba*I and *Xba*I/*EcoR*I restricted PCR products were cloned simultaneously in pNPTS138-*upstream*-*gspL* at *BamH*I/*EcoR*I sites. Resulting pNPTS138-*upstream*-*mgfp*-*gspL* was introduced in CB15N by electroporation. Allelic replacement of *gspL* by *mgfp*-*gspL* was checked by colony PCR and DNA sequencing using primers GFP-GSPL-CHECKF / GFP-GSPL-CHECKR: CATTCCCGATCGAGCAGGGC / ACCACGACCAGCGCCGCAAC.

Allelic replacement of *elpS* by *elpS-3flag* was obtained by homologous recombination between CB15N and pNPTS138-*elpS*-*3flag*-*downstream*. Sequence coding for *elpS*-*3flag* was amplified with primers ELPS-3FLAG-1 /ELPS-3FLAG-2: CATGGGAGGATTCGTGAAGCTGTATAGAAACCT/CGACGAAGTCGCGTCGTCTTACTTGTCATCGTCATC from pMR10-*elpS*-*3flag*. Downstream region of *elpS* (500 bp) was amplified with primers ELPS-3FLAG-3/ELPS-3FLAG-4 : GATGACGATGACAAGTAAGACGACGCGACTTCGTCG/ ACTGAGAATTCACACGCTTCAGCGGCTCAGG. Resulting PCR products were mixed and used as a matrix for a second PCR with primers ELPS-3FLAG-1/ ELPS-3FLAG-4. Fused sequences of *elpS*-*3flag* and dowsteam region of *elpS* were cloned into pGEM-Teasy. Insert was checked by DNA sequencing with primers

UP-PGEMT/DO-PGEMT. EcoRI/EcoRI *elpS*-*3flag*-*aval* excised from pGEM-Teasy was cloned into pNPTS138. The resulting pNPTS138-*elpS*-*3flag*-*downstream* was introduced in CB15N by electroporation. Allelic replacement of *elpS* by *elpS*-*3flag* was checked by colony PCR with primers FLAG-INF /ΔELPS-CHECK-R: ATGGACTACAAAGAC /GTCAGCTTTCTTGTGCTGC.
